# Glances at the Hospitals

**Published:** 1901-08-24

**Authors:** 


### GLANCES AT THE HOSPITALS.

### THE LIGHTBURN JOINT HOSPITAL AT SHETTLESTON, LANARK.

This, the fourth annual report of the Fever Hospital, tells us that 41 patients were in residence on January 1st, 1899, and that 736 were admitted during the year. Of these 639 were discharged cured and 54 died, leaving 84 under treatment on December 31st. The average stay in the hospital, of recovered cases, was 46 days, and the average death rate for all diseases was 7·7 per cent. The scarlet fever cases numbered 561, and 480 of these were discharged cured and 23 died. The rest remained under treatment. The mortality from scarlet fever was 4·5 per cent., but this percentage would be reduced a little if one case dying within 24 hours be omitted, and another, a convalescent, dying from measles. One case sent in as typhus proved to be scarlet fever, and in three other cases the diagnosis was uncertain throughout. Dr. Fletcher states that the most marked feature of 1899 epidemics was the large proportion of anginous cases, and consequently causing a high death rate. Of the fatal cases 12 resulted from septicæmia, 4 from bronchitis, 2 from pneumonia, 1 from nephritis, 1 from œdematous laryngitis, 1 from hæmorrhage, and 1 from enteritis. The usual scarlatina complications occurred in the following numbers:—Inflammation of the middle ear in 41 cases, rhinorrhœa in 34, rheumatism in 22, inflammation of kidneys in 20, bronchitis in 13, cervical abscess in 13, pneumonia in 5, and cervical cellulitis in 1. During the year 149 cases of enteric fever were admitted. The average stay in the hospital (recovered cases) was 43 days. The death rate was 16·5 per cent., but two deaths took place within 48 hours. It is curious to notice that the death rate among the men was twice as high as it was among the women. The diphtheria cases numbered 15. Of these 12 recovered and 3 died. The staff of this hospital consists of one resident physician, one matron, two charge nurses, two trained nurses, nine probationers, a serving maid, a cook, two housemaids, three laundry maids, two ward maids, and one kitchen maid. The report is very clearly written, and the statistics can be followed with ease; but we hope that next year Dr. Fletcher will include a table showing the cost of maintenance of patients and staff. Such information could not fail to be most valuable at present when fever hospitals are springing up near so many of our large towns.

